# 亲和分离在蛋白质泛素化修饰研究中的应用进展

**DOI:** 10.3724/SP.J.1123.2020.07005

**Published:** 2021-01-08

**Authors:** Huifei ZHONG, Yanyan HUANG, Yulong JIN, Rui ZHAO

**Affiliations:** 1.北京分子科学国家研究中心, 中国科学院活体分析化学重点实验室, 中国科学院分子科学科教融合卓越创新中心, 中国科学院化学研究所, 北京 100190; 1. Beijing National Laboratory for Molecular Sciences, CAS Key Laboratory of Analytical Chemistry for Living Biosystems, CAS Research/Education Center for Excellence in Molecular Sciences, Institute of Chemistry, Chinese Academy of Sciences, Beijing 100190, China; 2.中国科学院大学, 北京 100049; 2. University of Chinese Academy of Sciences, Beijing 100049, China

**Keywords:** 蛋白质泛素化, 亲和色谱, 抗体, 多肽, protein ubiquitination, affinity chromatography (AFC), antibody, peptide

## Abstract

蛋白质泛素化是真核生物最普遍、最复杂的翻译后修饰方式之一,在细胞的信号转导、生长、发育、代谢等生命过程中发挥着重要作用。泛素化过程的失调则与神经退行性疾病、炎症反应、癌症等重大疾病的发生发展密切相关。分析和研究蛋白质泛素化的结构与功能,可望为认识生命、探索疾病调控内在规律和发现新的诊断策略提供重要信息。生命体系的高度复杂性,泛素化修饰位点、结构类型的多变和多样性,时空动态变化等特点给蛋白质泛素化分析研究带来了巨大的挑战。亲和分离以其高选择性成为泛素化蛋白质结构与功能研究的有力工具。免疫亲和分离法基于抗原-抗体相互作用,是最为经典的分离分析方法,已广泛应用于泛素化蛋白质或肽段的富集分离。源于天然泛素受体的泛素结合结构域(ubiquitin binding domains, UBDs)可与泛素或多聚泛素链相互作用。UBDs和基于此发展起来的串联泛素结合实体(tandem ubiquitin-binding entities, TUBEs)已成为蛋白质泛素化功能研究的热门识别分子。各种多肽类化合物的发展也为蛋白质泛素化的结构和功能解析提供新工具。此外,多种亲和识别配基的联合使用,在蛋白质泛素化修饰的高特异性、高灵敏度分析中展现了独特的优势,为认识生命体内的泛素化修饰提供了重要保障。该文对亲和分离方法在蛋白质泛素化修饰分析中的应用及进展进行了综述。

## Abstract (en)

Protein ubiquitination is one of the most common yet complex post-translational modifications in eukaryotes that plays an important role in various biological processes including cell signal transduction, growth, and metabolism. Disorders in the ubiquitination process have been revealed to correlate with the occurrence and development of many diseases such as neurodegenerative disease, inflammation, and cancer. Investigation of protein ubiquitination is of great importance to uncover protein functions, understand the molecular mechanisms underlying biological processes, and develop novel strategies for disease treatment. Great advances have been made toward understanding protein ubiquitination; however, it remains a challenging task due to the high diversity of ubiquitination sites and structures, as well as the dynamic nature of ubiquitination in biological processes. Protein ubiquitination occurs through the formation of a covalent bond between the carboxyl terminus of ubiquitin and the ε-amino group of a lysine residue in the substrate. As a small protein, ubiquitin itself can be further modified by another ubiquitin molecule to form homotypic or heterotypic polyubiquitin chains. There are eight sites, namely seven lysine residues (K6, K11, K27, K29, K33, K48, and K63) and one N-terminal methionine (M1), in one ubiquitin molecule that can be used to form a ubiquitin dimer. The variations in modification sites, ubiquitin chain lengths, and conformations result in differences in protein sorting, cell signaling, and function. To resolve the high complexity of protein ubiquitination, new separation approaches are required. Affinity separation based on the specific recognition between biomolecules offers high selectivity and has been employed to study the structures and functions of ubiquitination. In addition, affinity ligands are central to the separation performance. Different affinity ligands have been developed and employed for the capture and enrichment of ubiquitylated proteins. Immunoaffinity separation based on antigen-antibody interactions has been one of the most classical separation methods. Antibodies against ubiquitin or different ubiquitin linkages have been developed and widely applied for the enrichment of ubiquitylated proteins or peptides. The specific capture allows the downstream identification of endogenous ubiquitination sites via mass spectrometry and thus facilitates understanding of the roles and dynamics of polyubiquitin signals. Ubiquitin-binding domains (UBDs) are a collection of modular protein domains that can interact with ubiquitin or polyubiquitin chains. Ubiquitin-associated domains, ubiquitin-interacting motifs, and ubiquitin-binding zinc finger domains are the most frequently used UBDs. Due to the moderate affinity of UBDs toward ubiquitin or ubiquitin chains, tandem ubiquitin-binding entities (TUBEs) have been engineered with high affinities (*K*_d_ in the nanomolar range) and exhibit potential as powerful tools for ubiquitination analysis. Because of their affinity and selectivity, UBDs and TUBEs have been applied for the isolation and identification of ubiquitylated targets in cancer cells and yeasts. Compared with antibodies and UBDs, peptides are smaller in size and can be facilely synthesized via chemical approaches. The modular structure of peptides allows for *de novo* design and screening of artificial ubiquitin affinity ligands for targeted capture of ubiquitinated proteins. Furthermore, the polyhistidine tag at the N-terminus of ubiquitin facilitates the purification of ubiquitylated substrates using immobilized metal affinity chromatography. Considering the high complexity of biosystems, strategies combining multiple affinity ligands have emerged to further improve separation efficiency and reduce background interference. Several combinations of antibodies with UBDs, antibodies with peptidyl tags, and UBDs with peptidyl tags have been developed and proven to be effective for the analysis of protein ubiquitination. These affinity-based approaches serve as important solutions for studying the structure-activity relationship of protein ubiquitination. This review highlights the applications and recent advances in affinity separation techniques for analyzing protein ubiquitination, focusing on the methods using antibodies, UBDs, peptides, and their combinations as affinity ligands. Further, their applications in the enrichment of ubiquitin-modified substrates and the identification of ubiquitination structures are introduced. Additionally, remaining challenges in affinity separation of protein ubiquitination and perspectives are discussed.

将蛋白质贴上泛素分子(ubiquitin, Ub)标签,即泛素化,是真核生物最普遍、最复杂的翻译后修饰方式之一,参与并调控着包括蛋白质降解、DNA修复和信号转导等诸多生理过程^[1,2,3]^。泛素化过程的失调或紊乱与癌症、阿尔兹海默症、亨廷顿病等人类重大疾病密切相关^[4,5,6]^。可见,全面而深入认识蛋白质泛素化修饰,对于蛋白质新功能的揭示,疾病靶标的发现和化学干预具有重要作用。泛素化过程是一个涉及多种酶的级联催化反应。通过将泛素分子C端羧基连接到底物蛋白质的赖氨酸(Lys, K)残基的ε-氨基上,即可实现蛋白质的泛素化修饰^[7,8]^(见图1a)。泛素化修饰过程复杂,产物、位点、形式多样,给蛋白质泛素化的功能解析和调控研究带来了巨大的挑战。不同于磷酸化、甲基化等小分子修饰基团,泛素分子自身是一个含有76个氨基酸的小蛋白,其中N-端甲硫氨酸(Met1, M1)自由氨基和7个赖氨酸残基(K6、K11, K27、K29、K33、K48、K63)的侧链氨基可以作为反应位点继续被修饰,形成不同长度、不同结构的泛素链,传递不同的信号,执行不同的功能(见图1a)。K48泛素链主要参与蛋白质经蛋白酶体的降解过程,K63泛素链在DNA损伤修复、信号转导、免疫应答等过程发挥作用,M1泛素链是炎症信号通路的关键调节因子,K11泛素链参与细胞周期的调控,K6泛素链和线粒体的质量控制相关,K27泛素链调节先天性免疫应答过程,K29和K33泛素链参与多种激酶的修饰^[1-3,9-11]^。此外,泛素还可进一步进行磷酸化等其他多种类型的翻译后修饰^[12]^,进一步增加了蛋白质泛素化的多样性和复杂性(见图1b)。

**图 1:**
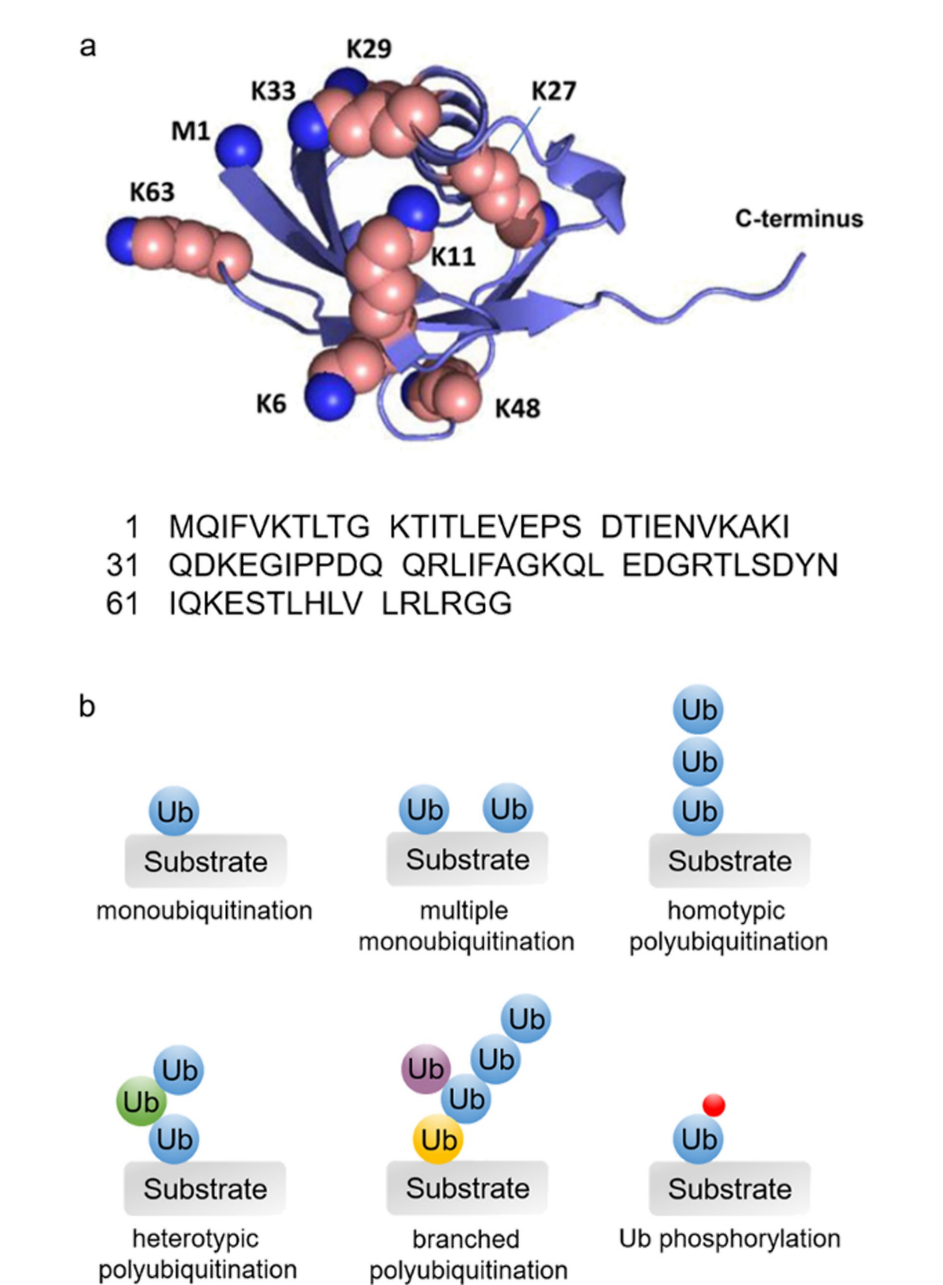
(a)泛素的氨基酸序列、三维结构和修饰位点^[8]^与(b)泛素化修饰类型示意图

生命分离分析新原理和新方法为蛋白质泛素化结构和功能探测提供了重要突破口。亲和分离技术是基于生物分子之间特异性相互作用发展而来的,广泛用于亲和色谱、样品前处理等领域^[13]^。近年来,以相应的泛素化修饰为靶标,构建高亲和力、高专一性的亲和配基,发展亲和分离新方法,成为蛋白质泛素化修饰分析研究的有力工具^[8,14]^。与此同时,质谱技术的高速发展为泛素化蛋白质的高通量鉴定提供了保障^[15,16,17]^。将亲和分离和液相色谱-质谱联用技术结合,是泛素化修饰通路研究中不可或缺的方法^[18]^,为揭示蛋白质泛素化修饰的生物学效应、探索疾病调控规律提供重要信息。本文针对亲和分离新材料和新方法在蛋白质泛素化修饰分析中的应用进展进行了综述。

## 1 免疫亲和分离法用于泛素化修饰分析

免疫亲和分离是基于抗原-抗体特异性相互作用进行目标分子分离的方法。抗体作为最经典的生物识别分子,具有亲和力高、选择性好的特点,在蛋白质泛素化的分离分析中发挥了至关重要的作用^[19]^。例如,泛素分子的单克隆抗体既可特异性识别单泛素化产物,也能识别多泛素化缀合物,已广泛应用于泛素化蛋白质的分离富集^[20]^。此外,K48、K63和M1等泛素链的特异性抗体也被用作特定泛素链的亲和识别配基,在相关泛素化功能研究中发挥作用^[21,22,23]^。

泛素化蛋白质经酶解后,产生的非泛素化肽段对泛素化位点的检测产生严重干扰。为了促进蛋白质泛素化位点的高通量鉴定,美国哈佛大学-麻省理工学院Broad研究所的Udeshi等^[24]^以抗赖氨酸-ε-甘氨酸-甘氨酸(Lys-ε-Gly-Gly, K-ε-GG)抗体为亲和配基,将其化学交联在蛋白A琼脂糖珠表面,制备了新型分离材料,用于泛素化肽段的富集。泛素分子C端存在精氨酸-甘氨酸-甘氨酸(Arg-Gly-Gly, RGG)三肽片段,其中C末端甘氨酸的羧基通过异肽键连接到底物蛋白质的赖氨酸残基的ε-氨基上,由此形成特征的K-ε-GG标签,可被K-ε-GG抗体特异性识别(见图2)。因此,含K-ε-GG抗体的琼脂糖珠可用于蛋白质泛素化修饰的特异性分析。利用该方法,实现了细胞和组织样本中泛素化肽段的亲和分离,结合LC-MS/MS鉴定到了10000个泛素化位点。

**图 2:**
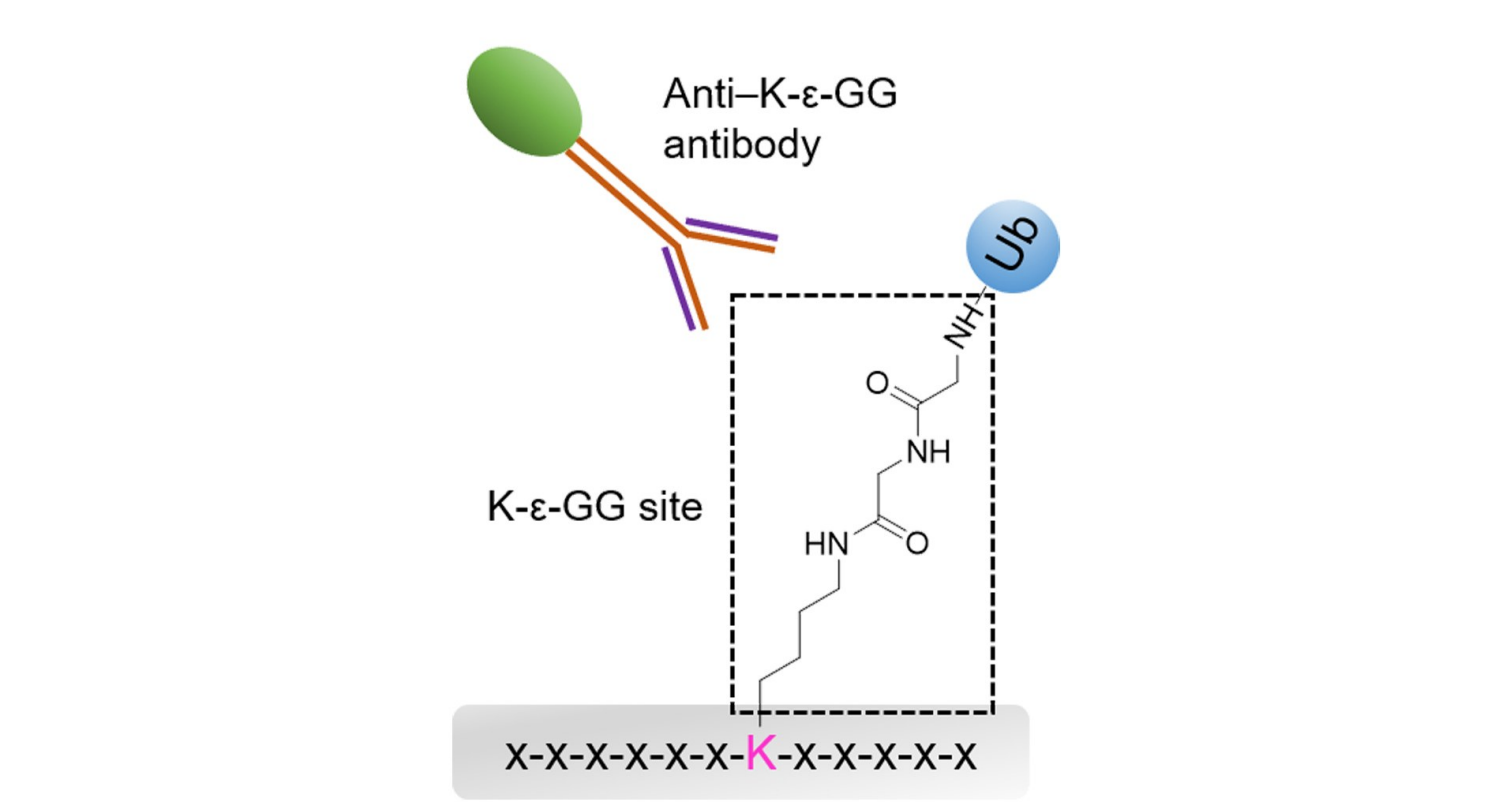
蛋白质泛素化修饰位点K-ε-GG的结构

如前所述,泛素化修饰种类多样复杂,且泛素自身也会被进一步修饰,因此亟需特异性更高的抗体作为识别元件。虽然抗K-ε-GG抗体在泛素化鉴定中发挥了重要作用,研究发现神经前体细胞表达下调因子8(neural precursor cell expressed developmentally down regulated 8, NEDD8)、干扰素刺激基因15(interferon-stimulated gene 15, ISG15)等类泛素分子也会对蛋白质进行修饰,NEDD8或ISG15的底物蛋白质经酶切后也会产生K-ε-GG结构,从而干扰泛素化位点的鉴定。为了解决这些问题,Akimov等^[14]^以泛素分子C端独有的13肽(ESTLHLVLRLRGG)为靶标,制备了一种新型的单克隆抗体UbiSite。当泛素化蛋白质经赖氨酸C端内切酶(LysC)水解后,泛素分子C端的13个氨基酸被保留在底物肽段上,因而可被UbiSite特异性识别。利用UbiSite为配基的亲和分离材料,他们实现了不同组织来源细胞样本中泛素化肽段的富集分离,结合LC-MS/MS鉴定到了63455个泛素化位点,对应9207个泛素化蛋白质,同时鉴定了104个N末端泛素化蛋白质,发现N末端泛素化和乙酰化之间呈负相关,为相关的生物学研究提供了重要信息。

## 2 基于UBDs亲和识别的泛素化修饰分析

泛素结合结构域(ubiquitin binding domains, UBDs)一般由20~150个氨基酸组成,与泛素或多聚泛素链存在特异性相互作用^[25]^。目前已鉴定出近25个不同亚家族的UBDs,包括泛素相关结构域(ubiquitin-associated domain, UBA)、泛素相互作用基序(ubiquitin-interacting motifs, UIMs)、锌指(zinc finger, ZnF)家族的泛素结合锌指结构域(ubiquitin-binding zinc finger domain, UBZ)等^[12]^。其中一些UBDs对特定泛素修饰方式具有专一性识别能力(见图3),如:蛋白hHR23A的UBA可优先结合K48泛素链;受体相关蛋白80(receptor-associated protein 80, RAP80)中的UIMs可特异性识别K63泛素链^[8,9]^。以这些UBDs为亲和配基,制备分离材料,在蛋白质泛素化修饰研究中具有重要的应用价值。

**图 3:**
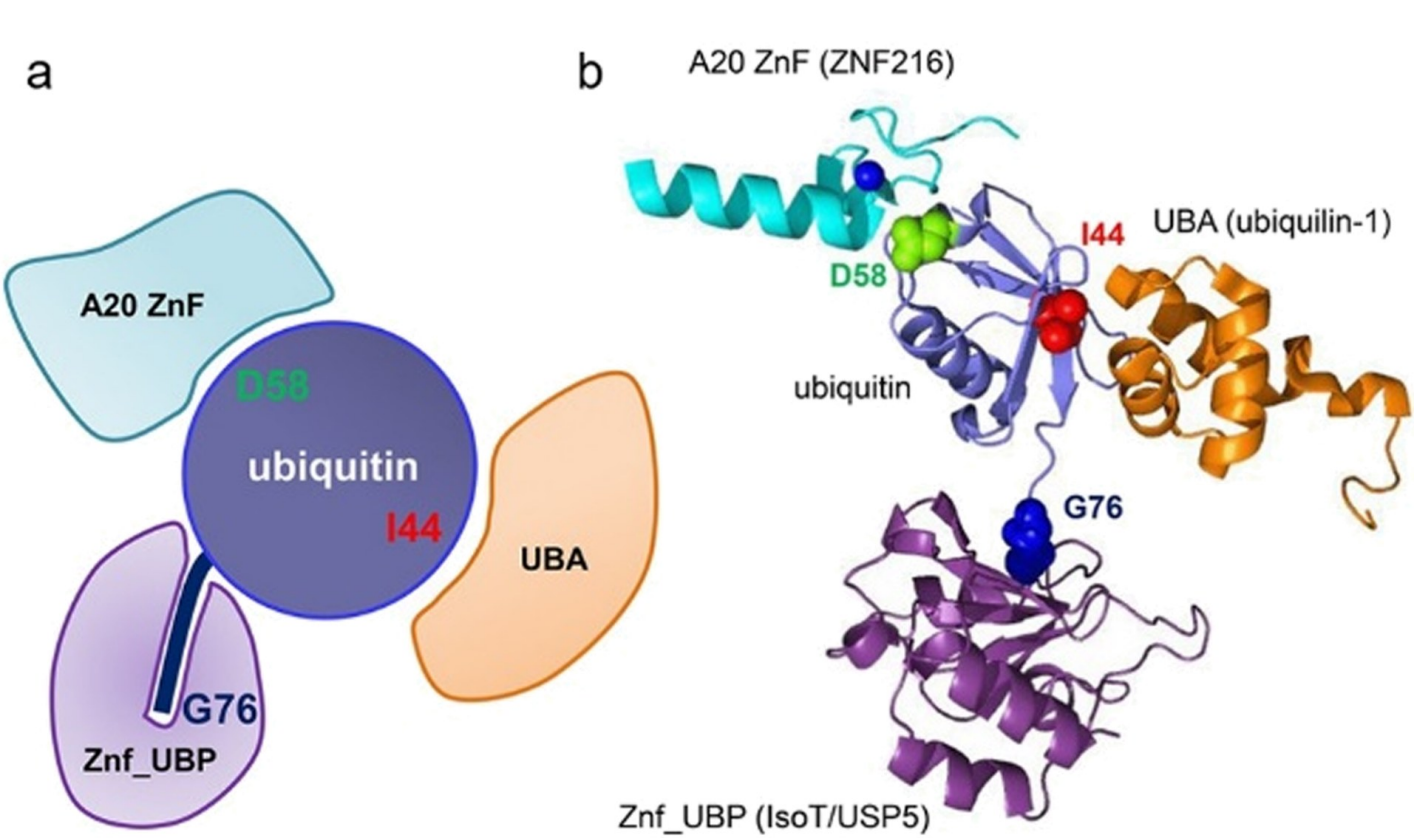
(a)UBDs的识别位点与(b)UBDs-泛素复合体的结构模型^[8]^

随着蛋白质泛素化研究的日益深入,基于UBDs的亲和分离法面临一些挑战。和抗体相比,多数UBDs和泛素分子之间亲和力弱(*K*_d_=10~500 μmol/L);同时UBDs对不同类型的泛素链具有亲和力差异,难以全面反映蛋白质的泛素化水平。为了获得具有普适性的UBDs,提高泛素化蛋白质的分离效率,Gao等^[26]^利用多个对不同泛素链具有结合力的UBDs为识别配基,以聚甘氨酸为柔性连接分子,构建了串联杂交泛素结合结构域(Tandem hybrid UBDs, ThUBDs)。ThUBDs由2~4个混合型UBDs单元串联而成,有利于多位点同时识别,可实现对7种不同泛素链的高效结合,其中ThUBDs和K48泛素链的*K*_d_值可达4.46 μmol/L。结合该亲和分离方法,实现了高转移人肝癌细胞中泛素化蛋白质的分离纯化;结合LC-MS/MS技术,成功鉴定了1125个泛素化蛋白质。

泛素链的长度往往通过分析其凝胶电泳的迁移率来确定。底物蛋白质上泛素化修饰位点的多样性为不同泛素链的长度测定提出了巨大的挑战。Tsuchiya等^[27]^通过串联UBA片段,构建了可特异性识别多聚泛素链的串联泛素结合实体(tandem ubiquitin-binding entities, TUBEs),建立了一种泛素链长度分析新方法Ub-ProT(Ub chain protection from trypsinization)。TUBEs和泛素链的结合可保护泛素化蛋白质不受去泛素化酶(deubiquitinases, DUBs)和蛋白酶体的降解,保持了完整的结构特性,进而可用电泳对其长度进行简单快速分析。该研究团队将Ub-ProT应用于酵母裂解物中底物蛋白质上泛素链的长度测定,发现K11和K63泛素链主要以二聚体形式存在,而K48、K6和K29泛素链中泛素分子多达7个;揭示了哺乳动物细胞中表皮生长因子受体(epidermal growth factor receptor, EGFR)泛素修饰特点,其中K63连接的泛素链以四聚体至六聚体为主。

## 3 基于多肽识别的泛素化修饰分析

多肽是重要的内源性生理活性物质,参与调节生物体内众多的生理过程^[28,29]^。和抗体等生物大分子相比,多肽具有相对分子质量小、分子相互作用快、可控性强、制备简便稳定等特点。针对细胞、蛋白质等靶标,人工设计合成靶向多肽,已成为化学和生物学关注的热点,也为复杂生命体系的分离分析提供了识别工具^[30,31,32,33,34,35,36,37]^。在泛素分子末端加多聚组氨酸标签(His_6_-tag),再利用固定化金属离子亲和色谱(immobilized metal affinity chromatography, IMAC)进行泛素化蛋白质的分离、富集或纯化^[38]^,是多肽识别在泛素化修饰分析中的一个重要应用。镍离子固定化亲和色谱分离主要在变性条件下进行,有效降低了DUBs对泛素化蛋白质的水解活性,同时可避免蛋白-蛋白相互作用引起的非泛素化蛋白质的结合,在蛋白质泛素化的分离分析中具有独特优势^[13,39]^。

由直链多肽环化形成的环肽具有亲和力高、选择性好、抗生物降解等优势,在特定泛素链的高效分离中具有应用前景^[40,41]^。以色列理工学院的Nawatha等^[42]^分别以K48泛素二聚体(^K48^Ub_2_)和泛素四聚体(^K48^Ub_4_)为靶标,利用mRNA展示技术获得随机肽库,筛选得到了靶向环肽Ub2i、Ub2ii、Ub4i及Ub4ix。利用表面等离子体共振(surface plasmon resonance, SPR)及^1^H-、^15^N-NMR分析了环肽和K48泛素链的相互作用。结构分析表明,Ub2ii和^K48^Ub_2_的作用位点位于泛素分子由亮氨酸、异亮氨酸和缬氨酸形成的疏水表面,结合比为1:1, Ub4ix则同时结合^K48^Ub_4_的前3个泛素分子。其中3种环肽(Ub2i、Ub2ii和Ub4i)对^K48^Ub_2_及^K48^Ub_4_均具有高结合能力,亲和力可达nmol水平;环肽Ub4ix则可特异性识别^K48^Ub_4_, *K*_d_值低至6 nmol/L。针对不同结构泛素链设计筛选亲和多肽作为识别配基,为蛋白质泛素化特异性分析提供了新工具。

相比于K48、K63修饰泛素链的相对高丰度,K6、K29和K33泛素链在细胞内含量极低,并且缺乏特异性识别分子。剑桥大学的Michel等^[43]^以K6和K33泛素链为靶标,通过多肽噬菌体展示技术,筛选出了K6和K33泛素链的多肽Affimer,测定了Affimer和K6、K33、K11泛素链的结合热力学和动力学特性。其中,K6 Affimer可特异性识别K6泛素链(*K*_d_<1 nmol/L), K33 Affimer对K33和K11泛素链均具有高效结合能力(见图4)。他们将K6 Affimer应用于人胚胎肾细胞中泛素化蛋白质的亲和分离,结合绝对定量法和bottom-up分析方法,不仅实现了K6泛素链的高效富集,而且鉴定了泛素连接酶HUWE1;揭示了HUWE1介导线粒体融合蛋白2(mitofusin2, Mfn2)上K6泛素链的修饰功能。

**图 4:**
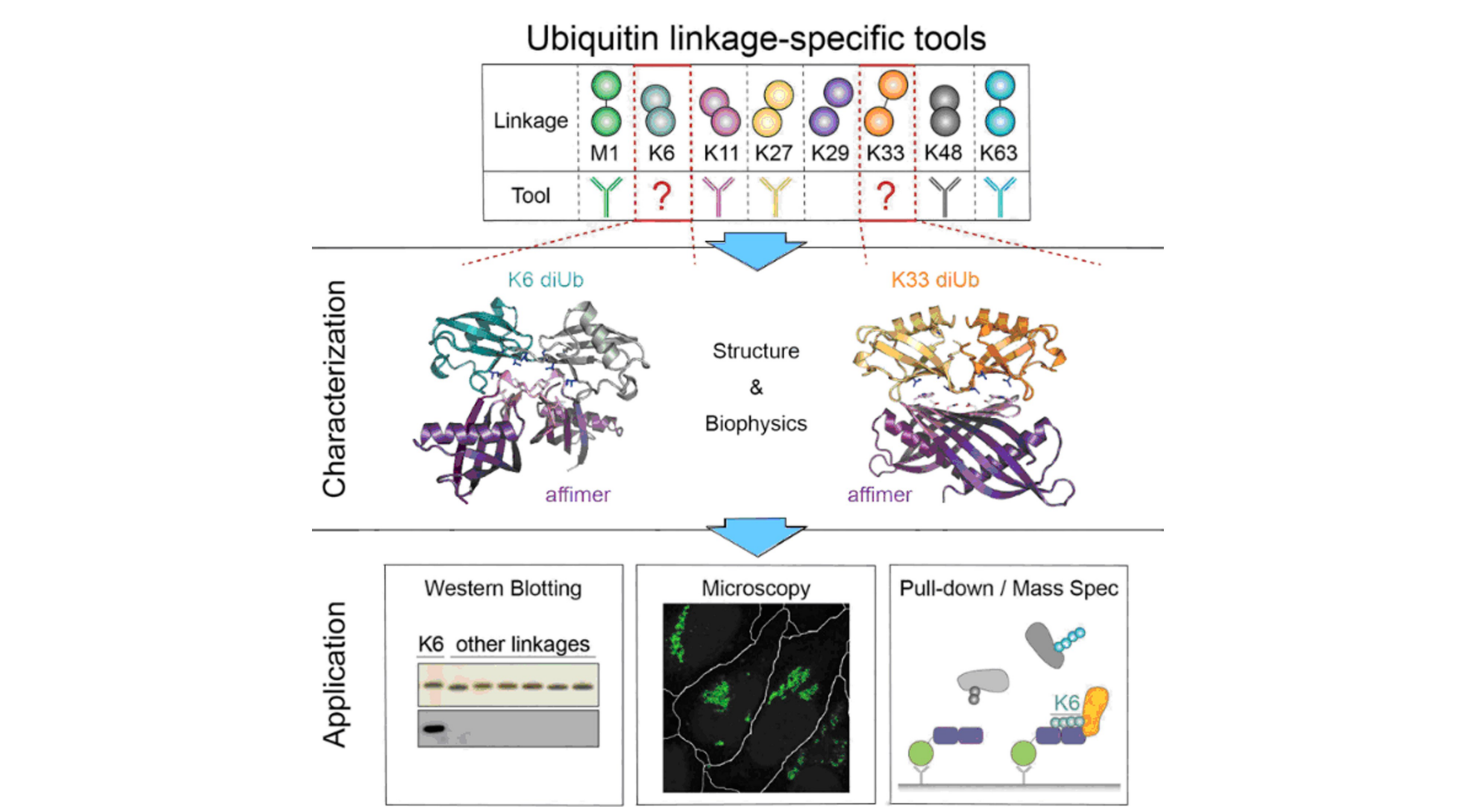
多肽Affimer的表征和应用^[43]^

## 4 基于多种亲和配基的泛素化修饰分离分析

细胞内泛素化蛋白质的含量普遍很低。通过单一亲和配基直接分离复杂生物样品中的泛素化蛋白质或肽段,分离效果仍比较有限,尤其是一些非典型的、丰度极低的泛素化修饰类型,很难实现高效富集。为了降低背景干扰,提高亲和分离效率,利用多种配基进行串联或并联识别,可望实现蛋白质泛素化的高效、高灵敏度分离分析。例如,将生物素-链霉亲和素识别体系(*K*_d_=10^-15^ mol/L)与His_6_-金属离子识别相结合,研究人员构建了新型串联亲和标签:组氨酸-生物素(histidine-biotin, HB)^[44]^。携带HB标签的泛素化蛋白质可在变性条件下,被镍亲和树脂、链霉亲和素琼脂糖捕获,从而实现分离纯化。基于该方法,在酵母细胞中分离鉴定到了258个泛素化蛋白质。密歇根大学的Maine等^[45]^利用泛素分子N端的His_6_标签,结合底物蛋白C端的生物素标记,建立了一种双分子亲和纯化法。镍亲和色谱的初次分离,可得到所有泛素化蛋白质;后续链霉亲和素的再次纯化,可去除其他非目标蛋白质的干扰,最终得到所需的泛素化靶蛋白。该方法被应用于3种特定泛素化靶蛋白的分离,纯度高达95%。

联合使用抗体和UBDs作为亲和配基,进行蛋白质泛素化的识别和分离分析,也是一种行之有效的方法。为了实现泛素连接酶相关底物蛋白质的高效鉴定,Yoshida等^[46]^利用蛋白Ubiquilin1的UBA片段,构建了携带FLAG标签的TUBEs。TUBEs对8种泛素链均具有结合能力,可防止底物蛋白的去泛素化和降解。在TUBEs对泛素链识别的基础上,利用抗FLAG抗体和抗K-ε-GG抗体进行两步亲和纯化,实现了底物蛋白泛素化肽段的高效分离富集。结合LC-MS/MS方法,鉴定到了连接酶FBXO21的底物蛋白质,可望成为泛素连接酶功能研究的有效手段(见图5)。

**图 5:**
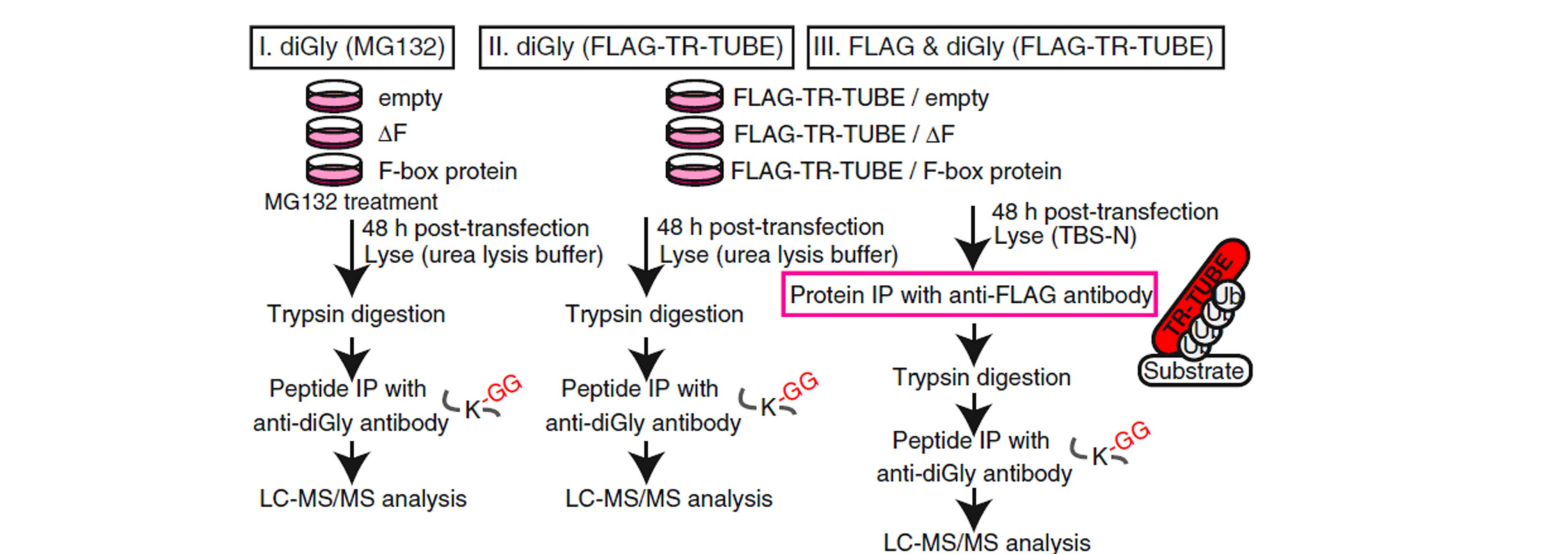
底物鉴定过程的示意图^[46]^

一系列酶促级联反应执行蛋白质泛素化过程。如何高效分离并鉴定泛素连接酶特异性底物蛋白质,对于认识泛素化过程具有重要意义。Mark等^[47]^通过泛素连接酶和UBA片段的融合,设计了携带FLAG标签的连接酶陷阱(ligase trap)。UBA和泛素链的结合,有利于提高连接酶对其泛素化底物的亲和力。针对连接酶陷阱上的FLAG标签和泛素分子上的His_6_标签,以抗FLAG抗体和镍离子为识别单元,建立了串联亲和纯化方法,实现了酵母和哺乳动物细胞中泛素连接酶相关底物蛋白的亲和分离;结合质谱技术,鉴定到了多种新型底物蛋白。

类泛素化修饰(small ubiquitin-like modifier, SUMO)与泛素化修饰密切相关,但研究两种修饰之间相互关系的分析方法仍比较有限。为了同时鉴定蛋白质泛素化和SUMO化修饰,McManus等^[48]^突变SUMO分子C端肽段序列(QQQTGG)为RNQTGG,因此SUMO化修饰蛋白质酶解后将产生含有K(NQTGG)结构的肽段,可被抗K(NQTGG)抗体特异性识别;同时构建了携带His_6_标签的SUMO突变体(SUMO3m)。通过上述分子设计,利用镍亲和树脂分离得到了SUMO化修饰蛋白质,富集倍数高达100倍;继而利用抗K(GG)抗体及抗K(NQTGG)抗体进行免疫沉淀,实现了泛素化肽段和SUMO化肽段的亲和分离。基于高的富集倍率和选择性,结合LC-MS/MS技术,仅需16 mg细胞提取物,即可鉴定8000多个SUMO化修饰位点,以及3500多个泛素化修饰位点,为阐明两种蛋白质修饰之间的功能和联系提供了关键技术手段。

## 5 总结与展望

基于生物分子间特异性相互作用的亲和分离技术,在蛋白质泛素化修饰分析中发挥了举足轻重的作用。一些天然存在的识别配基,如:抗体、UBDs和生物素等,已成功用于泛素化修饰蛋白质的分离富集之中。与此同时,人工设计筛选新型识别分子(如:靶向多肽),也得到了关注,并为泛素化修饰的特异性分析提供了新工具。不仅如此,多种亲和识别配基的联合使用,拓宽了泛素化蛋白质高选择性分析的思路,降低了复杂生物体系中的背景干扰,提高了分离富集的效率。不同亲和分离方法可望为蛋白质泛素化结构与功能的认识提供新工具,在药物新靶标的发现、治疗干预等方面具有应用潜能。

然而蛋白质泛素化修饰的研究仍存在诸多挑战。一些细胞内含量极低的非典型泛素化修饰(如:K6、K27、K29和K33泛素链),其功能尚未完全揭示;和同型泛素链相比,混合型泛素链和分支型泛素链结构更为复杂,对异型泛素链的识别和功能研究仍非常欠缺。因此,针对这些泛素化分子特征,构建新的亲和识别分子,建立高效、高选择性的亲和分离方法,对于满足这些低丰度、可能具有关键生物功能的泛素链的分离分析具有非常重要的意义,也是未来研究关注的方向。
